# ICTV Virus Taxonomy Profile: Tospoviridae 2026

**DOI:** 10.1099/jgv.0.002353

**Published:** 2026-10-30

**Authors:** Paolo Margaria, Scott Adkins, Ozgur Batuman, Amalendu Ghosh, Holly R. Hughes, Rayapati A. Naidu, Massimo Turina, Anna E. Whitfield

**Affiliations:** 1 Plant Virus Department, Leibniz Institute DSMZ-German Collection of Microorganisms and Cell Cultures GmbH, Braunschweig, Germany; 2 United States Department of Agriculture, Agricultural Research Service, U.S. Horticultural Research Laboratory, Fort Pierce,Florida, USA; 3 Plant Pathology Department, University of Florida, Immokalee, Florida, USA; 4 Insect Vector Laboratory, Advanced Centre for Plant Virology, ICAR-Indian Agricultural Research Institute, New Delhi, India; 5 Centers for Disease Control and Prevention, Fort Collins, Colorado, USA; 6 Department Plant Pathology, Irrigated Agriculture Research and Extension Center, Washington State University, Prosser, WA, USA; 7 Institute for Sustainable Plant Protection, National Research Council of Italy, Brescia, Italy; 8 Department of Plant Protection, School of Agriculture, The University of Jordan, Amman, Jordan; 9 Department of Entomology and Plant Pathology, North Carolina State University, Raleigh, NC, USA

**Keywords:** ICTV Report, *Orthotospovirus*, taxonomy, Tospoviridae

## Abstract

*Tospoviridae* is a family of plant viruses with genomes consisting of three molecules of negative-sense or ambisense ssRNA, comprising about 17 kb in total. Virions are enveloped, with spherical or pleomorphic morphology, and display surface projections composed of two viral glycoproteins. Arthropods in the family Thripidae serve as biological vectors for transmission to plants. Members of the family are responsible for economically important diseases in vegetable, field and ornamental crops. This is a summary of the International Committee on Taxonomy of Viruses Report on the family *Tospoviridae*, which is available at ictv.global/report/tospoviridae.

## Virion

Virions are spherical or pleomorphic, 80–120 nm in diameter, and composed of a phospholipid membrane with glycoprotein surface projections (Table 1, Fig. 1). The core contains the genomic RNAs assembled into ribonucleoprotein (RNP) complexes together with the nucleocapsid protein (N) and an RNA-directed RNA polymerase (RdRP). Within individual virus particles, the number, identity, completeness and polarity of segments are widely variable [1].

**Table 1.** Characteristics of members of the family *Tospoviridae*

Example	tomato spotted wilt virus (L: D10066; M: S48091; S: D00645), species *Orthotospovirus tomatomaculae*
Virion	Enveloped, spherical or pleomorphic, 80–120 nm in diameter
Genome	Negative-sense or ambisense ssRNA in three linear segments of about 8.9, 4.8 and 2.9 kb
Replication	Cytoplasmic
Translation	From capped mRNAs that lack a poly(A) tail
Host range	Plants, invertebrates
Taxonomy	Realm *Riboviria*, kingdom *Orthornavirae*, phylum *Negarnaviricota*, subphylum *Polyploviricotina*, class *Bunyaviricetes*, order *Elliovirales*: the family includes the genus *Orthotospovirus* and >35 species

**Fig. 1.**
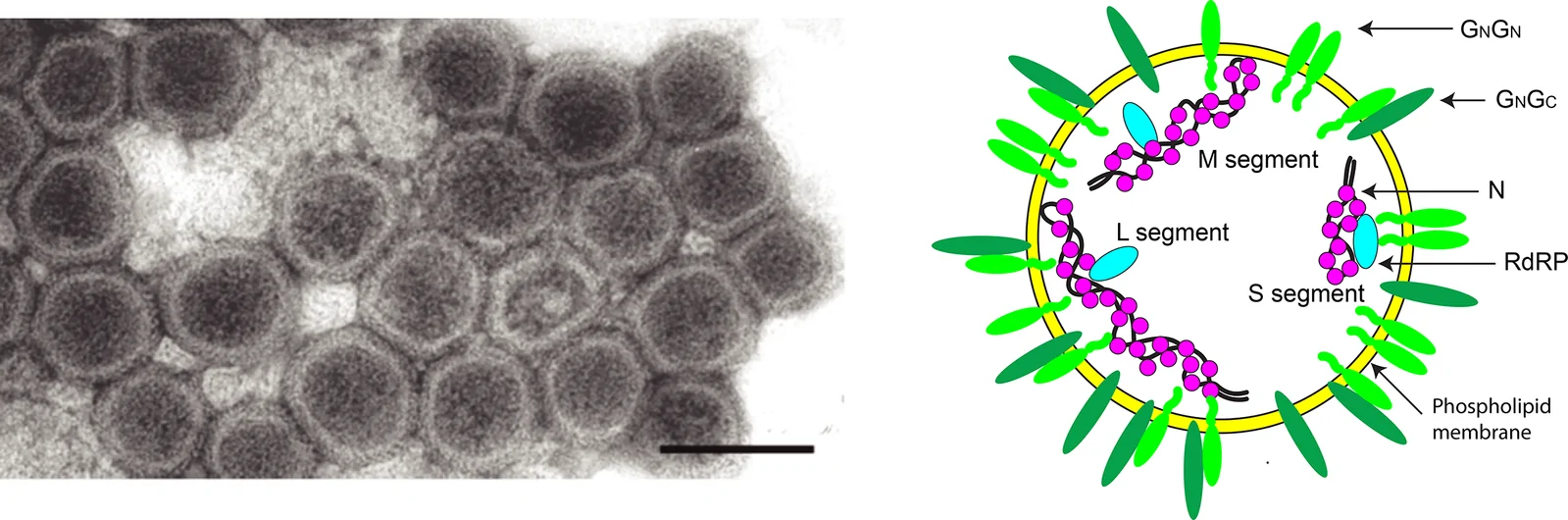
(left) Electron micrograph of negatively-stained particles of tomato spotted wilt virus (bar: 100 nm, courtesy of Dr Jan van Lent) and (right) virion graphical representation (N, nucleoprotein; RdRP, RNA-directed RNA polymerase; G_N_, G_C_, N-terminal and C-terminal glycoproteins).

## Genome

The genome consists of three negative-sense or ambisense RNA molecules, of about 17 kb in total, termed the large (L), medium (M) and small (S) RNA, with complementary sequences at the 5′- and 3′-termini and coding for a large protein (L, including an RdRP domain), the G_N_ and G_C_ envelope glycoproteins, the nucleocapsid protein (N) and two non-structural proteins (NSm, NSs) [2] (Fig. 2).

**Fig. 2.**
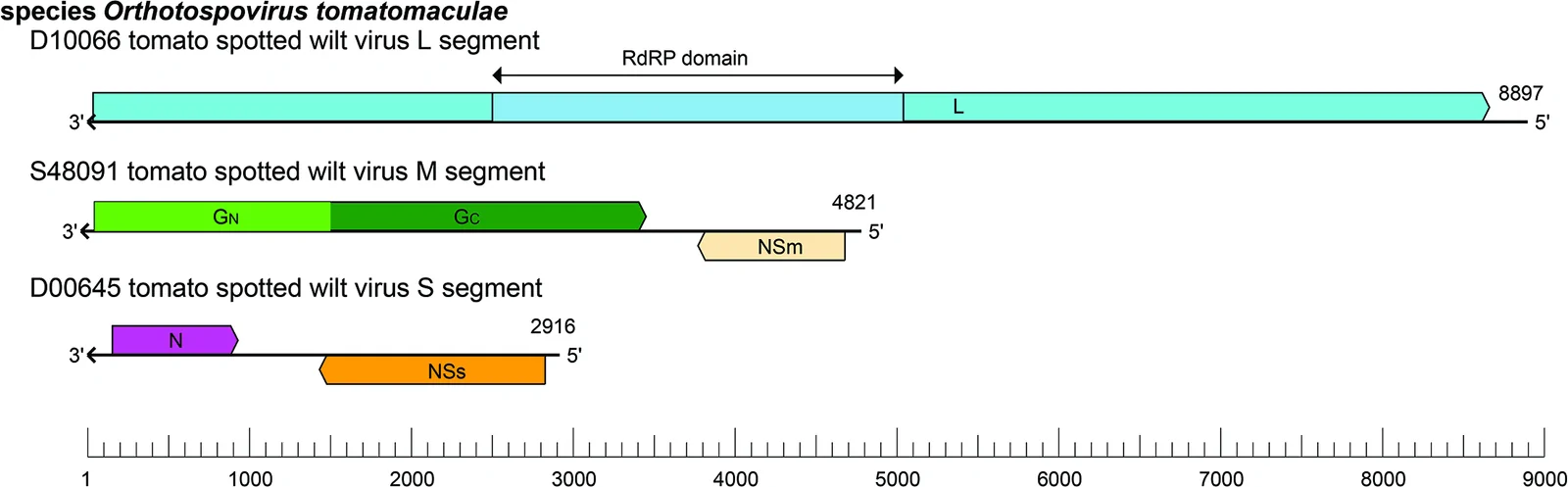
Genome organization of tomato spotted wilt virus, a representative member of the family *Tospoviridae*.

## Replication

Viral mRNAs lack a 3′-poly(A) tail and are generated through a cap-snatching mechanism whereby the viral transcriptase complex cleaves mature host cellular mRNAs ~12–18 nucleotides downstream of the 5′-cap and utilizes the resulting capped oligonucleotides as primers for transcription [3]. Genome replication occurs in electron-dense cytoplasmic inclusions. Newly synthesized RNP complexes either traffic intra- and inter-cellularly or mature into enveloped virus particles through coordinated multimeric interactions of the three major structural proteins (N, G_N_ and G_C_) at the endoplasmic reticulum and Golgi membranes. Mature virions accumulate within large intracellular vesicles [4], and are acquired by thrips during feeding. Replication also occurs in the insect vector.

## Pathogenicity

Members of the family infect a wide range of plants including tomato, pepper, tobacco, peanut, soybean, lettuce and potato [2], inducing chlorosis, necrosis, ringspots, wilting and stunted growth. Transmission is by thrips (Thysanoptera: Thripidae). Natural genetic resistance sources against orthotospoviruses are available [5]; emergence of resistance-breaking isolates of tomato spotted wilt virus is of global concern.

## Taxonomy

Current taxonomy: ictv.global/taxonomy. Species in the only genus (*Orthotospovirus*) are defined on the basis of vector specificity, plant host range, serological relationships of the N protein and <90% identity of the N protein sequence with that of any other described species in the genus.

## Resources

Full ICTV Report on the family *Tospoviridae*: ictv.global/report/tospoviridae.

## Abbreviations

N: nucleocapsid protein
RdRP: RNA-directed RNA polymerase
RNP: ribonucleoprotein
